# The novel application of artificial neural network on bioelectrical impedance analysis to assess the body composition in elderly

**DOI:** 10.1186/1475-2891-12-21

**Published:** 2013-02-06

**Authors:** Kuen-Chang Hsieh, Yu-Jen Chen, Hsueh-Kuan Lu, Ling-Chun Lee, Yong-Cheng Huang, Yu-Yawn Chen

**Affiliations:** 1 Research Center, Charder Electronic Co., LTD, Taichung, Taiwan; 2 Department of Radiation Oncology, Mackay Memorial Hospital, Taipei, Taiwan; 3 Sport Science Research Center, National Taiwan University of Physical Education and Sport, Taichung, Taiwan; 4 Graduate Institute of Sport Coaching Science, Chinese Culture University, Taipei, Taiwan; 5 Department of Physical Education, National Taiwan University of Physical Education and Sport, 16, Sec. 1, Shuan-Shih Rd, Taichung, 40404, Taiwan

**Keywords:** Back Propagation Artificial Neural Network (BP-ANN), Body composition, Bioelectrical impedance analysis (BIA), Elderly, Dual-energy X-ray absorptiometry

## Abstract

**Background:**

This study aims to improve accuracy of Bioelectrical Impedance Analysis (BIA) prediction equations for estimating fat free mass (FFM) of the elderly by using non-linear Back Propagation Artificial Neural Network (BP-ANN) model and to compare the predictive accuracy with the linear regression model by using energy dual X-ray absorptiometry (DXA) as reference method.

**Methods:**

A total of 88 Taiwanese elderly adults were recruited in this study as subjects. Linear regression equations and BP-ANN prediction equation were developed using impedances and other anthropometrics for predicting the reference FFM measured by DXA (FFM_DXA_) in 36 male and 26 female Taiwanese elderly adults. The FFM estimated by BIA prediction equations using traditional linear regression model (FFM_LR_) and BP-ANN model (FFM_ANN_) were compared to the FFM_DXA_. The measuring results of an additional 26 elderly adults were used to validate than accuracy of the predictive models.

**Results:**

The results showed the significant predictors were impedance, gender, age, height and weight in developed FFM_LR_ linear model (LR) for predicting FFM (coefficient of determination, r^2^ = 0.940; standard error of estimate (SEE) = 2.729 kg; root mean square error (RMSE) = 2.571kg, *P* < 0.001). The above predictors were set as the variables of the input layer by using five neurons in the BP-ANN model (r^2^ = 0.987 with a SD = 1.192 kg and relatively lower RMSE = 1.183 kg), which had greater (improved) accuracy for estimating FFM when compared with linear model. The results showed a better agreement existed between FFM_ANN_ and FFM_DXA_ than that between FFM_LR_ and FFM_DXA._

**Conclusion:**

When compared the performance of developed prediction equations for estimating reference FFM_DXA_, the linear model has lower r^2^ with a larger SD in predictive results than that of BP-ANN model, which indicated ANN model is more suitable for estimating FFM.

## Background

Body composition is routinely measured to evaluate the nutritional status of patients in clinical setting. The prognosis of morbidity and mortality in the elderly are strongly associated with nutritional status [1,2]. In the elderly, the fat mass (FM) decreases with age [3] and differences in gender become prevalent [4]. The assessment results of body composition can be used to prevent malnutrition, monitor health risks, design physical therapy programs, facilitate the improvement of heath programs [5] and predict drug kinetics in the elderly [6]. Therefore, the accuracy and precision of the measuring results in the elderly will be critical in clinical application.

Currently, many body composition measurements are limited in their applications to the elderly. The non-invasive, simple, safe, fast and inexpensive properties of bioelectrical impedance analysis (BIA) make this method an applicable measurement for the elderly [7]. The measurement of body composition using BIA oftentimes includes many predictive variables, such as impedance, ethnicities, age, sex, height and weight to develop linear prediction equations for estimating body fat content [8].

Despite the fact that the standing hand-to-foot BIA is more convenient than the supine hand-to-foot BIA [9], the standing hand-to-foot BIA has not yet been widely used except for limited reports in the current research literature [10]. The simple operational procedure for conducting a standing hand-to-foot BIA measurement can efficiently measure body composition in clinical application and epidemiological researches [11]. The impedance measured by BIA can incorporate with other predictive variables, such as age, sex, activity levels and ethnicities to develop a prediction equation, if the estimated results are validated by DXA can provide a relatively accurate estimation of body composition, especially using standing hand-to-foot BIA method [12]. Furthermore, some populations possess specific physiological characteristics such as the obese subjects [13], adolescents [14], young women with high physical activity levels [15] and elite male athletes [16] may require a specific developed BIA prediction equation for obtaining more accurate estimates. The existing published BIA equations were developed through linear regression analysis by using independent variables such as height, weight, sex, age and impedance [7]. The above rationale assumed that the relationship between the independent variables and dependent variable exhibits a linear relationship rather than non-linear relationship [17].

The linear regression model was used to describe the relationship between a single dependent variable such as FFM and other independent variables such as impedance, height, age, weight and sex. While the linear regression model may appear to be simple and applicable; however, when choose several variables as predictors to construct a multivariable regression model which may violate the basic assumption about independence of explanatory variables from one another. Since anthropometric variables often correlated with each other, the colinearty can lead to mistaken conclusions. Therefore, the linear regression model may not be a suitable method for developing a prediction equation. The results of previous BIA studies in elderly adults have shown that the association between anthropometric variables and body composition parameters were not very strong [18]; therefore, an improvement of prediction equation is needed.

Other prediction models, including logistic regression [19], Cox regression [20], discriminant analysis, recursive partitioning [21] and artificial neural network-ANN [22], have been widely used in clinical applications for diagnosis [23], imaging [24], the analysis of wave forms [25], the identification of pathological specimens [26], clinical pharmacology [27] and outcome prediction [28,29]. Two studies had utilized the BIA measurements with an ANN model to evaluate the intracellular fluid [30] and total water body in patients under chronic hemodialysis [31]. The results of these two studies showed that ANN model performed better in predictive accuracy than a linear regression analysis did [30,31]. Very few studies have investigated the measurement of whole body composition, lean body mass and skeleton muscle mass using BIA measurement with ANN analysis. Whether the ANN model exhibits greater precision and accuracy in BIA measurement than the linear model is an interesting issue.

In the present study, we measured the FFM of Taiwanese male and female elderly adults using both BIA and DXA to develop a Back Propagation - Artificial Neural Network (BP-ANN) predictive model and compared the results with those of the linear predictive model to evaluate whether the ANN model exhibits greater accuracy.

## Methods

### Subjects

Healthy elderly subjects age 55 and over without chronic diseases such as hypertension, diabetes mellitus, cancer, nephrotic syndrome, hepatitis-related disease, chronic pulmonary disease, or artificial electrical implantation and assist devices, were recruited with the permission of the Institutional Review Board (IRB) of the Advisory Committee at Jen-Ai Hospital in Taiwan. 48 elderly males and 40 elderly females from central Taiwan were informed with formal consent forms prior test. The 62 randomly sampled subjects used to develop the BP-ANN mathematical model for the estimation of FFM were called the modeling group (MG), and the remaining 26 subjects comprised the validation group (VG).

### Experimental procedures

The body weight and height of the subjects were measured to the nearest 0.1 kg and 0.5 cm, respectively. All of the subjects were dressed in cotton robe without any metal attachments for the whole body DXA (Lunar Prodigy, GE Corp, USA.) measurements. The results were analyzed with “enCore 2003 Version 7.0” software. The whole body scanning protocol of each subject was completed within twenty minutes. All measurements were conducted by licensed technicians in the Radiology Department of the Dah Li County Jen-Ai Hospital in Taiwan. The FM and FFM were estimated by DXA. After DXA measurements, the subjects stood on a platform embedded with tetra-polar electrodes and gripped a handle embedded with bi-polar electrodes on the right hand side to measure the impedance at a frequency of 50 kHz. The impedance measurement instrument (QuadScan 4000; Bodystat, Ltd., Isle of Man, UK) contains independent detect electrodes and current source electrodes in the platform and handle grip. The total FFM values estimated by BIA using linear regression analysis (FFM_LR_) or by BIA using BP-ANN model analysis (FFM_ANN_) were compared to the DXA measurement (FFM_DXA_).

### Back propagation-artificial neural network (BP-ANN)

**Figure 1:**
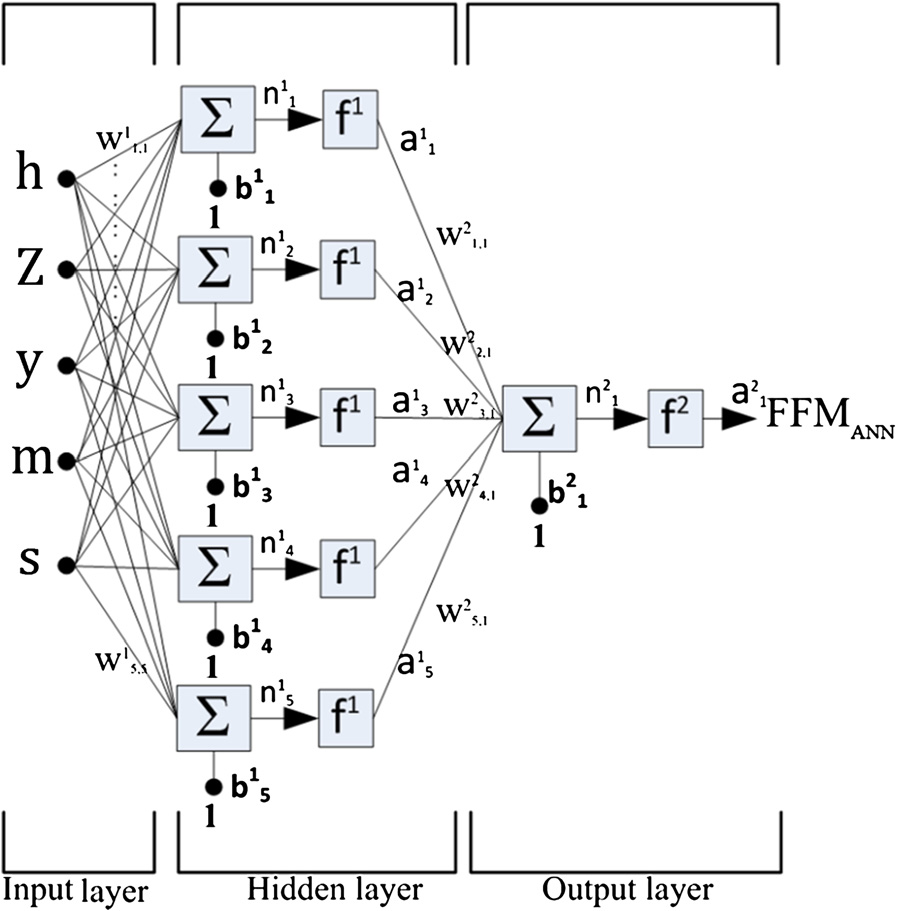
**The BP-ANN model used in present study included an input layer with 5 values, which included h, height; m, body weight; y, age; Z, impedances; s, gender; a hidden layer with 5 neurons and an output layer with one neuron.** The solid circles represent individual neurons and the lines represent the inputs, outputs and weighted connections between the neurons. **n**, net input; **b**^i^_j_, bias vector; **W**^i^_j,k_, weight matrix; **f**^i^, transfer functions; **a**^i^_j_, neuron output. The superscript i represents the serial number of layers and the subscripts j and k represent the serial number of the neuron and the input.

We created the FFM predicting model using the BP-ANN (Figure 1), including an input layer, hidden layer and output layer [32]. The input layer contained **p**_**j**_ (j =1 to 5) values, including height (h), weight (m), age (y), impedance (Z) and sex (S). The hidden layer contained the one to multiple neurons that combine both the **W**^1^_i,j_ (weight matrix) and **b**^1^_i_ (bias vector). In other words, the calculation of the input value using both the **W**^1^_i,j_ and **b**^1^_i_ gave the **n**^1^_i_ value, which was subsequently substituted into **f**^1^ (transfer function), which is the Log-Sigmoid function, to determine the **a**^1^_i_. The **a**^1^_i_ was termed the first hidden layer. The above equations can be expressed as follows:

(1) $$\begin{array}{ll} a^{1}{}_{i} & =f^{1}\left(W^{1}{}_{i,j}p_{j}+b^{1}{}_{i}\right)\\ & =f^{1}\left(n^{1}{}_{i}\right)=\mathrm{logsin}\left(W^{1}{}_{i,j}p_{j}+b^{1}{}_{i}\right) \end{array}$$

logsin (n) = 1/(1 + *e*^*-n*^)

Scalars – small *italic* letters

Vectors – small **bold** non-italic letters

Matrices – capital **BOLD** non-italic letters

i-the series number of the neuron

j-the number of input values (**p**_1_ = h, **p**_2_ = w, **p**_3_ = y, **p**_4_ = Z, **p**_5_ = s)

The outcome value **a**^**1**^ is connected to the output layer, which contains **f**^2^ (Linear transfer function). The above equation can be expressed as follows:

(2) $$\begin{array}{ll} a^{2} & =f^{2}\left(W^{2}{}_{i,1}a^{1}{}_{i}+b^{2}{}_{1}\right)=f^{2}\left(n^{2}{}_{1}\right)\\ & =\mathrm{purelin}\left(W^{2}{}_{i,1}a^{1}{}_{i}+b^{2}{}_{1}\right) \end{array}$$

purelin(n) = n

The output layer with a single hidden layer in the present BP-ANN model can be expressed as follows:

(3) $$\begin{array}{ll} a^{2}{}_{1} & =f^{2}\left(W^{2}{}_{i,1}f^{1}(W^{1}{}_{i,j}p_{j}+b^{1}{}_{i}\right))+b^{2}{}_{1})\\ & =\;\mathrm{purelin}\left(W^{2}{}_{i,1}\mathrm{logsin}(W^{1}{}_{i,j}p_{j}+b^{1}{}_{i}\right)+b^{2}{}_{1})\\ & \times \left(i=1\mathrm{to}5,j=1\mathrm{to}5\right) \end{array}$$

During the first training procedure, all of the anthropometric **p**_**j**_ values, which contain height, weight, age, sex and impedances, in the input layer were randomly weighted for each equation in the initial weight matrix as **W**^1^_i,j_ , **W**^2^_i,1_,with the addition of the initial values in the bias vector as **b**^1^_i_ , **b**^2^_1_. The target **t** FFM values were measured by a DXA. After comparing to the target **t** values, the network applied the Levenberg-Marquardt algorithm to optimize the bias vector and weight matrix, subsequently processing the data backward to repeatedly adjust the weight matrix and bias vectors until convergence. For the training rule in the present study, we set the maximum iteration as 1000 times, with a minimum gradient value of 10^-6^. All of the algorithms mentioned above were coded by Matlab Ver.7.0 (MathWorks, Inc. MA, USA). The BP-ANN models containing one to five neurons were created in the hidden layer to explore the effects of neuron number on the precision of FFM prediction. After the training process, the optimal weight matrix of the **W**^1^_i,j_ and **W**^2^_i,1_variables and the bias vector of the **b**^1^_i_ and **b**^2^_1_variables were obtained.

### Statistical analysis

All of the data were analyzed by SPSS version14.0 software (SPSS Inc., Chicago, IL, USA). The data are presented as the means ± standard deviation (SD). The data of 62 randomly sampled subjects were used to develop the BP-ANN model and linear regression model for predicting FFM. Multivariable linear regression was used to develop a linear FFM prediction equation for comparison with the ANN equation. The FFM_LR_ and FFM_ANN_ were compared with each other by using Bland and Altman plots in which the predictive results in each subject by both equations were plotted against reference FFM_DXA_; the differences in predicting BF% were also compared. The standard error of estimate (SEE) and root-mean-square error (RMSE) were also used to measure the accuracy of predictions. The coefficient of determination (r^2^) were calculated to compare the goodness of fit between two models. Also, the data of an additional 26 subjects were used to validate the developed equations. For all statistical analyses, a P value of < 0.05 was considered significant.

## Results

The basic characteristics and body composition data for the MG and VG are shown in Table 1. The mean age of the males and females in the MG group was 61.0 ± 5.14 years and 61.2 ± 5.8 years, respectively, while the mean body fat content of the male and female subjects was 27.0 ± 5.3% and 35.8 ± 6.7%, respectively. The mean age of the males and females in the VG group was 65.1 ± 5.0 years and 61.3 ± 5.07 years, respectively, while the mean body fat content was 27.0 ± 5.3% and 35.8 ± 6.7%, respectively.

**Table 1:** The basic characteristics and body composition data of the subjects

	**Mean ± SD**	**Range**	**Mean ± SD**	**Range**
**M.G.**^**1**^	**Male**	**(*n*** **=36)**	**Female**	**(*n*** **= 26)**
Age (years)	60.99±5.14	55.0, 71	61.2±5.8	55.0, 74.8
Height (m)	1.69±0.08	1.50, 1.91	1.57±0.06	1.46, 1.76
Weight (Kg)	73.8±13.6	53.8, 114.4	61.8±9.2	42.0, 79.7
BMI (Kg/m^2^)	25.8±3.8	20.3, 36.8	25.0±3.9	17.9, 35.4
Impedance (ohm)	545.4±60.4	407.6, 774.3	639.8±61.8	479.2, 777.0
FFM_DXA_ (kg)^3^	52.7±9.3	28.0, 79.1	37.3±4.6	29.7, 44.9
FM_DXA_ (kg)^3^	21.1±7.9	4.5, 37.9	24.4±7.1	10.7, 38.3
BF%_DXA_ (%)^3^	28.2±8.0	6.2, 49.2	39.0±7.3	21.4, 50.7
**V.G.**^**2**^	**Male**	**(*n*** **=12)**	**Female**	**(*n*** **= 14)**
Age (years)	65.1±5.0	59.5, 74.8	61.33±5.07	55.5, 73.2
Height (m)	1.67±0.07	1.56,1.80	1.54±0.05	1.43, 1.61
Weight (Kg)	71.4±7.5	57.1, 84.0	56.91±9.60	55.50, 73.20
BMI (Kg/m^2^)	25.6±1.8	21.5, 28.4	24.03±3.56	17.94, 29.72
Impedance(ohm)	565.2±47.9	493.0, 641.3	621.3±46.6	583.3, 741.0
FFM_DXA_ (kg)^3^	50.9±3.5	46.0, 59.6	35.7±4.4	28.9, 44.5
FM_DXA_ (kg)^3^	19.2±5.0	7.9, 26.5	20.6±6.8	12.0, 33.5
BF%_DXA_ (%)^3^	27.0±5.3	14.1, 34.4	35.8±6.7	26.6, 47.8

^1^MG: Modeling group; ^2^VG: Validation group; ^3^FFM_DXA_, FM_DXA_ (fat free mass) and BF%_DXA_ (percentage of fat percentage) were measured by DXA.

The linear prediction equation was obtained by linear regression analysis, height (h), weight (m), age (y), sex (S, 1: male, 0: female) and impedances were set as independent variables, and the FFM measured by DXA was set as dependent variables.

(4) $$\begin{array}{l} {\mathrm{FFM}}_{\mathrm{LR}}\left(\mathrm{kg}\right)=7.104+2.433\;S+0.{719\;h}^{2}/Z+0.217\;m–0.183\;y\;\\ \left(r^{2}=0.940,\mathrm{standard}\;\mathrm{error}\;\mathrm{of}\;\mathrm{estimate}\left(\mathrm{SEE}\right)=2.729\;\mathrm{kg},P<0.001\right) \end{array}$$

**Figure 2:**
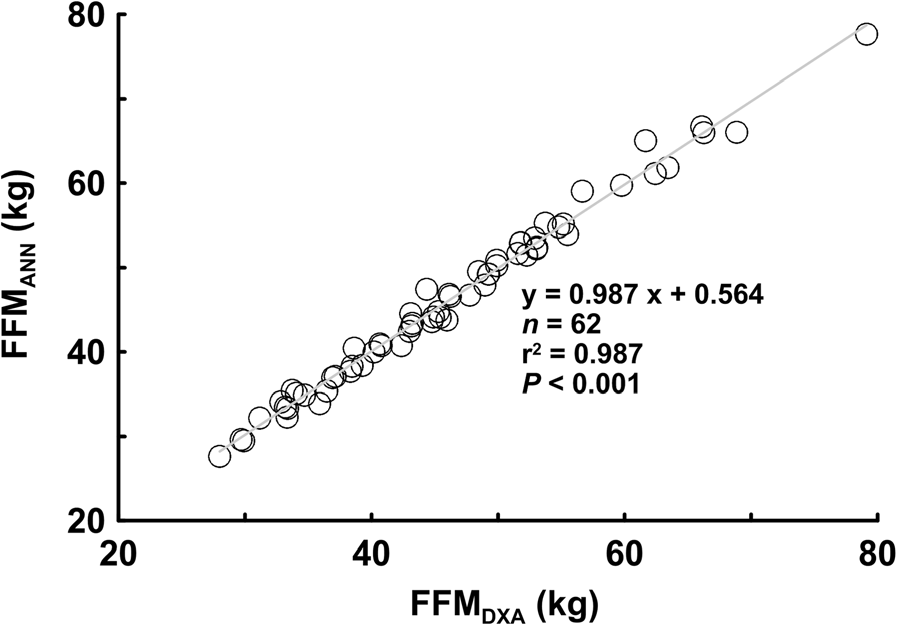
The relationship between the FFM values predicted by the BP-ANN model and the FFM values measured by DXA is shown for the modeling group.

During the training process, the hidden layers containing one to five neuron units in the BP-ANN model were executed with starting values of 1000 by the optimal algorithms (Levenberg-Marquardt (L-M) or Bayesian Regularization (B-R)) separately to obtain the optimal weight matrix **W**^1^_i,j_ , **W**^2^_i,1_ and bias vector **b**^1^_i_ , **b**^2^_1_. The **p**_**j**_ values were substituted into the optimal BP-ANN model to obtain the estimated FFM_ANN_ values. The effect of the number of neurons in the input layer on the determination coefficients of the FFM _DXA_ in the BP-ANN model is shown in Figure 2.

**Figure 3:**
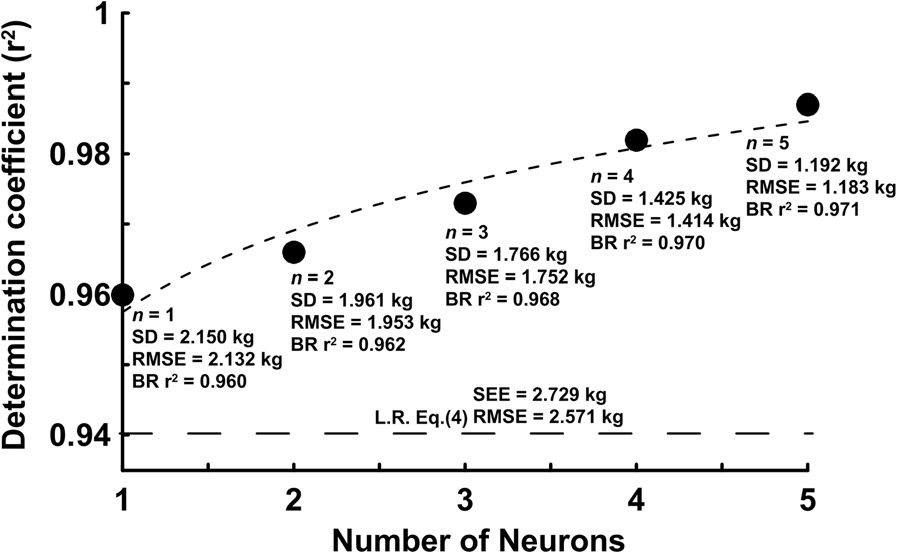
**The relationship between the number of neurons in the input layer of the BP-ANN models and the determination coefficients of the FFM values measured by DXA are shown for the modeling group.** SD, standard deviation; RMSE, root mean square error; BR r^2^, determination coefficients of predictive values by Bayesian Regularization and the FFM values measured by DXA.

The highest coefficients of determination (r^2^ = 0.987) occurred with five neurons in the predictive model; however, the highest coefficients of determination at one neuron unit still measured up to r^2^ = 0.960. We re-substituted the anthropometric and impedance values into the optimal BP-ANN model with five neurons to estimate the FFM_ANN_. The coefficient of determination of the estimated FFM_ANN_*vs.* FFM _DXA_ reached up to r^2^ = 0.987 with the L-M algorithm and r^2^ = 0.971 with the B-R algorithm (Figure 3).

**Figure 4:**
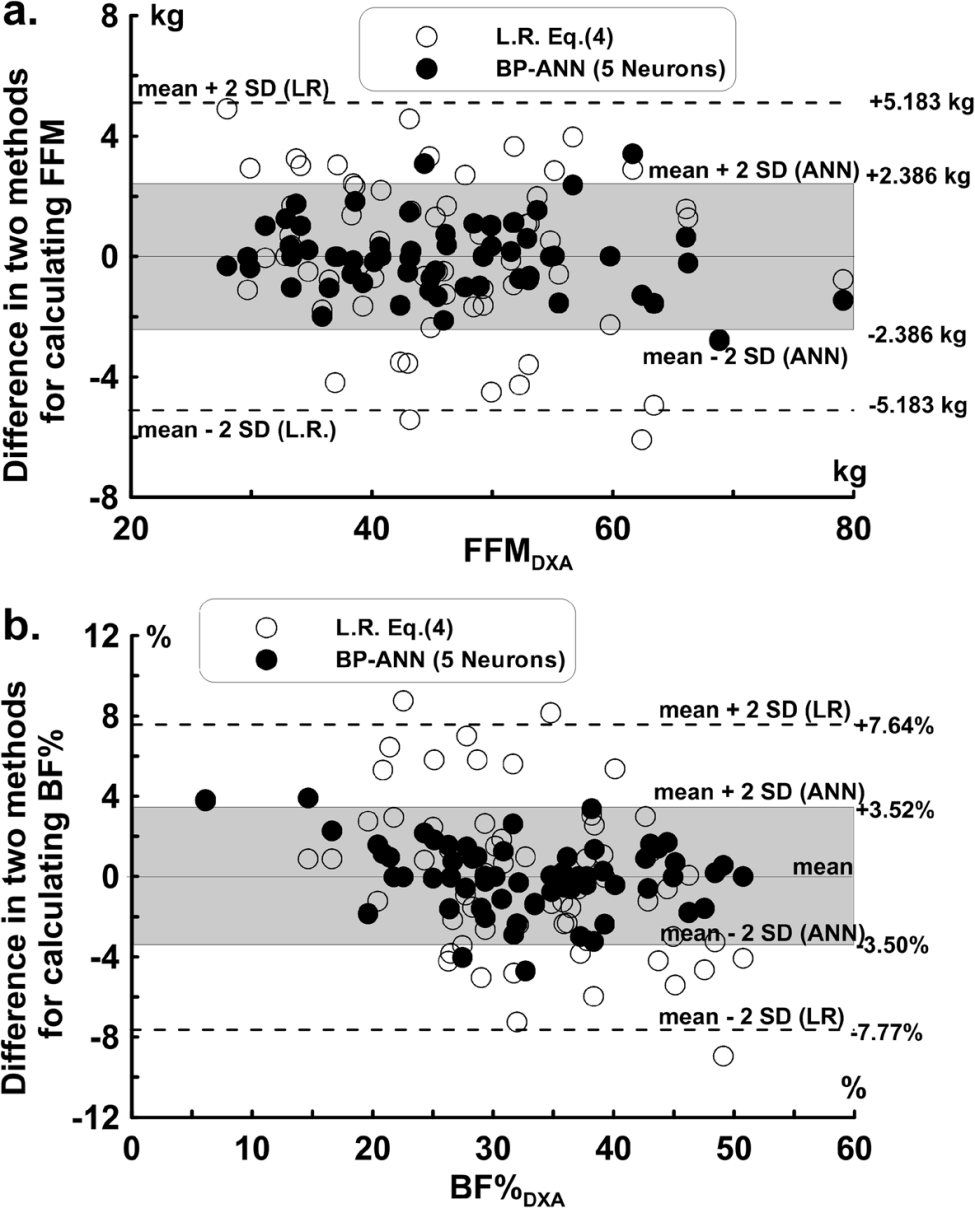
**The differences (bias) in the predicted (a) FFM and (b) BF% that are derived from both the linear prediction equations (LR) and the optimal BP-ANN (Back Propagation Artificial Neural Network) model are shown for the modeling group.** The empty circles represent the values predicted by the linear prediction equations (LR), and the solid circles represent the values predicted by the BP-ANN (Back Propagation Artificial Neural Network) model.

The Bland-Altman plot of bias in each predictive FFM value from both of the developed predictive equations is shown in Figure 4a The limits of agreement for estimated FFM_LR_ vs. FFM _DXA_ were ± 5.183 kg at 2 SD, while the limits of agreement for FFM_ANN_ vs. FFM_DXA_ was ± 2.386 kg at 2SD. The ranges of SEE in Eq. (4) (SEE = 2.729 kg) and in the optimal BP-ANN model (SD = 1.192 kg) are identified in Figure 4a The Bland-Altman plot of the differences between the body fat percentages estimated by both Eq. (4) and the optimal BP-ANN model against FFM_DXA_ is shown in Figure 4b. The SD of bias in Eq. (4) was 3.850%, while the SD of bias was 1.755% in the optimal BP-ANN model.

**Figure 5:**
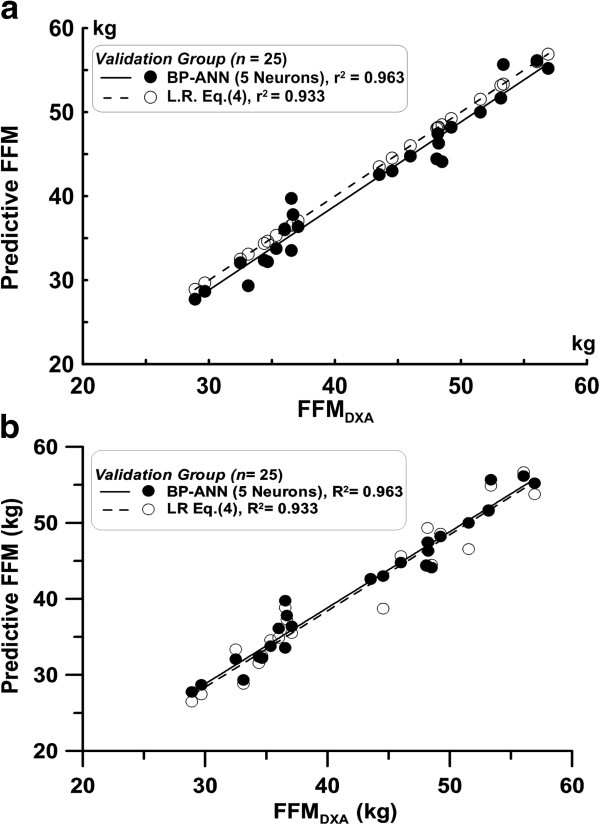
The relationship between the FFM predicted by the BP-ANN (Back Propagation Artificial Neural Network) model and the FFM values measured by DXA is shown for the validation group.

The FFM_LR_ and FFM_ANN_ estimated by the VG group vs. FFM_DXA_ analysis were 0.933 and 0.963, respectively. The above distributions are shown in Figure 5.

## Discussion

To elucidate the predictive performance in estimating the body composition for the elderly by using the linear model and the optimal BP-ANN model, identical dataset were used to develop these two models for comparison. Using the anthropometric data, the BP-ANN model with the simple input layer with five neurons was adopted to predict the FFM and body composition of the elderly. For predicting the FFM_DXA_, the coefficient of determination for the FFM_ANN_ (r^2^ = 0.960) estimated by the BP-ANN model with a single neuron in the input layer was greater than that of the FFM_LR_ estimated by the linear model (r^2^ = 0.940). The presence of more neurons in the input layer of the weighted BP-ANN model resulted in a higher coefficient of determination; the r^2^ value reached up to 0.987 when the five neurons were included in the input layer of the BP-ANN model. As more variables were included in the ANN model the correlation coefficient between predictive value and FFM_DXA_ increased, nearly approached to one. When compare the results with other studies using impedances in linear model, the FFM values for the elderly estimated by Genton et al. [33], Deurenberg et al. [34] and Roubenoff et al. [35] were underestimated approximately 2.9 to 7.1 kg in males and approximately 2.3 to 6.7 kg in females. Nevertheless, in comparison to the values determined by Baumgartner et al. [36], their results overestimated FFM roughly by 4.3 kg in males and approximately 1.4 kg in females. The data from Kyle et al. [37] show that the differences between the measured FFM and the DXA were 0.2 ± 2.0 kg in males and 0.0 ± 1.6 kg in females. Despite the acceptable coefficients of determination (r^2^ = 0.756-0.883) in the above-mentioned studies, improved r^2^ values were obtained in our five neurons input layer BP-ANN model. In particular, the smallest standard deviation of differences existed in the FFM_ANN_ vs. FFM_DXA_ comparison (0.0 ± 1.192 kg).

Because a larger computing capacity and longer processing time were required to exert the arbitrary function mapping or non-linear function mapping, we optimized the training process in BP-ANN model by using the Levenberg-Marquardt Algorithm to improve the convergence. Despite the limits of memory resources [32], the space required for our analysis is a relatively tiny amount in modern computer hardware. That trend makes our technique more applicable. To prevent the occurrence of a local error minimum in our BP-ANN model, we repeatedly applied various random initial values to the training process for the BP-ANN model. Meanwhile, the trial calculations for the errors and the correlation coefficient fit the optimal BP-ANN model.

With the same training data, the accuracy and precision of the BP-ANN model are directly related to the number of neurons and hidden layers. To prevent over-fitting in our BP-ANN model, the model was optimized by Bayesian Regularization. If the relationship between dependant variable and independent variables were linear, using BP-ANN model to develop linear prediction equation, with proper training similar or nearly identical results to linear regression may be achieved. However, if the relationship were non-linear, using linear regression model to construct prediction equation, the predictive accuracy will be limited [38]. When constructing a BP-ANN model, there was no guideline or rules for how many hidden layers should be constructed, how many neurons should be included, and how to choose proper transfer function for achieving the optimal predictive equation. For practical application, the different combination of layers and neurons may be used to construct model via training conjoin with validation analysis to achieve desired results. In most case, when the included hidden layers and neurons approach certain numbers, the estimated error will be minimized to certain value which cannot be reduced as more hidden layers and neurons are included into model. This phenomenon was observed as we constructed our model. For the minimum sample size, ANN model can generate better results than that of linear model when sample size is lower than 2000 [39]. But ANN model still has its downside, the estimated weight matrix, bias vector cannot have the same interence and interpretation as linear regression coefficient [40]. Another downside of ANN model is the complex calculation of the model which demand higher computation capability of measuring system or device, but recent development of computer hardware had made this obstacle easily be overcome which results in widely application of ANN model [41].

After ruling out other sources of dependent variability, the linear regression can easily describe the relationship between the single independent variable and the single major dependent variable. However, the linear regression does not work well in the systems with the dependent variables correlated with each others, especially in the complex human physiological system. Many variables, such as sex, age, physical activity, diet, genetics, weight and height, can affect body composition or have non-linear relationship among variables [18]. These variables may interact with each other to influence the estimation of body composition. In other words, the multiple dependent anthropometric variables may exhibit a coupled relationship rather than an independent linear relationship as assumed in a multiple linear regression model [42].

Consequently, the application of non-linear functions and other more flexible mathematic functions to describe the relationships between body composition parameter (fat free mass) and multiple variables requires much more attention to improve the predictive accuracy. In fact, the RMSE for FFM in our BP-ANN model was much lower than in LR model. Further evidence provided by Liu et al. shows that the application of the BIA system and the ANN model to estimating the FFM of the lower limbs exhibits greater performance than a linear model [43].

Many studies had successfully apply ANN model in clinical trials [22,24,27-31]. However, some indicated that ANN model can't perform better than linear regression model in clinical application. Therefore, the novel ANN model should be validated and use with care [39].

## Conclusions

Collectively, our study comparing the differences between the FFM_ANN_ and FFM_LR_ , the results of our study show superior outcomes with the BP-ANN model and indicate the successful application of this model in predicting the body composition of the elderly. The BP-ANN model may be incorporated into the measuring device for practical use in the future.

## Abbreviations

BIA: Impedance analysis; FFM: Fat free mass; BF%: body fat percentage; FM: Body fat mass; BP-ANN: Back propagation artificial neural network; FFM_LR_: FFM estimated with the analysis of linear regression model; FFM_ANN_: FFM estimated with the analysis of BP-ANN model; DXA: Dual-energy X-ray absorptiometry; Z: Impedance; FFM_DXA_: DXA measurement of FFM; H: Height; M: Weight; S: Sex; W^1^_i,j_: Weight matrix; b^1^_i_: Bias vector; f^1^: Transfer function; SD: Standard deviation; r^2^: Determination coefficient; SEE: Standard estimation of error; RMSE: Root mean square error; BR r^2^: Determination coefficients of estimation values by Bayesian Regularization and the FFM_DXA_; BF%_DXA_: DXA measurement of body fat percentage; BF%_LR_: BF% estimated by BIA with the analysis of linear regression model; FFM_ANN_: BF% estimated by BIA with the analysis of BP-ANN model.

## Competing interests

Charder Electronic Co., LTD funded this study. One author, Hsieh Kuen-Chang, belongs to the Research Center of the company. The other authors have declared that they have no competing interests.

## Authors’ contributions

KCH, the main author, contributed to the collection and interpretation of the BIA analysis data and developed the BP-ANN model; YJC completed the statistical analysis; YYC contributed to the collection and interpretation of the DXA analysis data and drafted the manuscript; HKL and YCH designed and revised the manuscript and assisted in the development of the BP-ANN model. All of the authors have read and approved the final manuscript.
